# Squamous cell carcinoma of the prostate: long-term survival after combined chemo-radiation

**DOI:** 10.1186/1748-717X-2-15

**Published:** 2007-04-03

**Authors:** Fernando Munoz, Pierfrancesco Franco, Patrizia Ciammella, Mario Clerico, Mauro Giudici, Andrea Riccardo Filippi, Umberto Ricardi

**Affiliations:** 1 Department of Medical and Surgical Sciences, Radiation Oncology Unit, University of Torino, Ospedale S. Giovanni Battista, Torino, Italy; 2 Department of Oncology, Medical Oncology, ASL 12, Biella, Italy; 3 Pathology Unit, ASL 12, Biella, Italy

## Abstract

**Background:**

Carcinoma of the prostate gland is the most frequent malignant tumour affecting male population. While the large majority of tumours is represented by adenocarcinoma, pure squamous cell carcinoma comprises only 0,5–1% of all prostate neoplastic lesions.

It is characterised by a high degree of malignancy, commonly metastasising to the bone (mainly with osteolytic lesions), liver and lungs with a median survival time of 14 months.

Several therapeutic approaches have been employed in the effort to treat prostate pure squamous cell carcinoma, including radical surgery, radiotherapy, chemotherapy and hormonal therapy. All of them mostly failed to gain a significant survival benefit.

**Case report:**

We herein report on a case of pure squamous cell carcinoma of the prostate approached with combined-modality treatment, with the administration of 3 courses of cisplatin 75 mg/m^2 ^on day 1 and continous infusion 5-fluorouracil 750 mg/m^2 ^on day 1 to 5 and, subsequently, radiotherapy, with the delivery of a total dose of 46 Gy to the whole pelvis, with additional boost doses of 20 Gy to the prostatic bed and adjunctive 6 Gy to the prostate gland (72 Gy in total). The patient remained free of disease for 5 years, finally experiencing local relapse and, subsequently, dying of acute renal failure due to bilateral uretero-hydro-nephrosis.

In addition, we provide a complete overview of all reported cases available within the medical literature.

**Conclusion:**

Since it remains questionable which should be the most appropriate therapeutic approach towards prostate pure squamous cell carcinoma, our report demonstrates that a prolonged disease control, with a consistent survival time, may be achieved by the combination of an effective local treatment such as radiotherapy with systemic infusion of chemotherapeutic drugs.

## Background

Carcinoma of the prostate gland is the most frequent malignant tumour affecting male population [1]. The large majority of prostate tumours is represented by the adenocarcinoma histotype (up to 95%), while small cell, squamous cell, transitional cell, signet ring cell, mucinous and mixed types carcinomas occurs in less than 2% of cases [2,3]. Pure squamous cell carcinoma (PSCC) comprises only 0,5–1% of all prostate carcinomas [4]. Therefore clinical manifestations, natural history, prognosis and treatment options can be found only in anecdotal descriptions. It usually occurs in the seventh decade of age, with symptoms of urinary obstruction (due to bladder outlet obstacle) or bone pain due to osseous metastases [5]. Deemed rather more malignant than adenocarcinoma, PSCC commonly metastasises to the bone (mainly with osteolytic lesions), liver and lungs with a median post-diagnosis survival time estimated to be 14 months [6]. From the diagnostic point, serum Prostate-specific antigen (PSA) and prostatic acid phosphatase (PAP) commonly show values within the normal range, even in a metastatic disease context. However, squamous cell carcinoma (SCC) antigen might be elevated, allowing a serologic monitoring of disease status [7]. While radical prostatectomy and radiation are the only potentially curative options for prostate adenocarcinomas, several therapeutic approaches have been employed in the management of PSCC of the prostate, such as radical surgery, radiotherapy, chemotherapy and hormonal therapy. All of them have not been able to obtain long-lasting objective responses, neither in terms of local control, nor in terms of systemic efficacy. We herein present a case of PSCC of the prostate treated with a combined chemo-radiotherapeutic approach, resulting in a prolonged disease-free survival of 5 years. Moreover, we provide a systematic overview of all reported cases available within the medical literature.

## Case report

In June 2001, a 76 years old male was referred to our institution hospital with clinical signs and symptoms of acute urinary retention, complaining of voiding difficulties during the previous 2 months. On catheterisation, 600 ml of urine could be rescued. Physical examination was unremarkable, except from digital rectal examination which disclosed an uneven swollen and enlarged prostate gland, of stony-hard consistency, with an irregular capsule profile. The patient had been healthy until the time of our observation. No history of radiation and hormonal therapy could be highlighted. Hence, a complete diagnostic work-up was planned.

Serum PSA and PAP showed in-range values. Transrectal ultrasound demonstrated hypoechoic lesions in the left peripheral zone of the prostate gland. Excretion urogram, urine cytology and urethro-cystoscopy were negative for malignancy, while bladder neck was slightly elevated on cystoscopy.

An abdominal and pelvic computed tomography (CT) scan was then performed, disclosing an irregularly enlarged prostate with a peripheral hypodense mass within, compressing the base of the bladder and disrupting the prostatic anatomy. No enlarged lymphnodes could be detected at any abdominal site.

On magnetic resonance imaging (MRI), the boundary between the transition zone and the peripheral zone of the prostate gland was unclear and the signal intensity had decreased in the left peripheral zone on T2-weighted images. Extra-capsular disease could be documented in at least 2 sites of the prostatic profile. No osseous spread could be observed at total body bone scan.

Sextant transrectal ultrasound-guided needle biopsy of the prostate was subsequently performed, with histological examination demonstrating nests and sheets of moderately differentiated squamous carcinomatous cells characterised by intercellular bridges. Focal areas presented with evidence of keratin pearl formation. No squamous metaplasia or transitional cell or adenocarcinomatous components could be observed.

**Figure 1:**
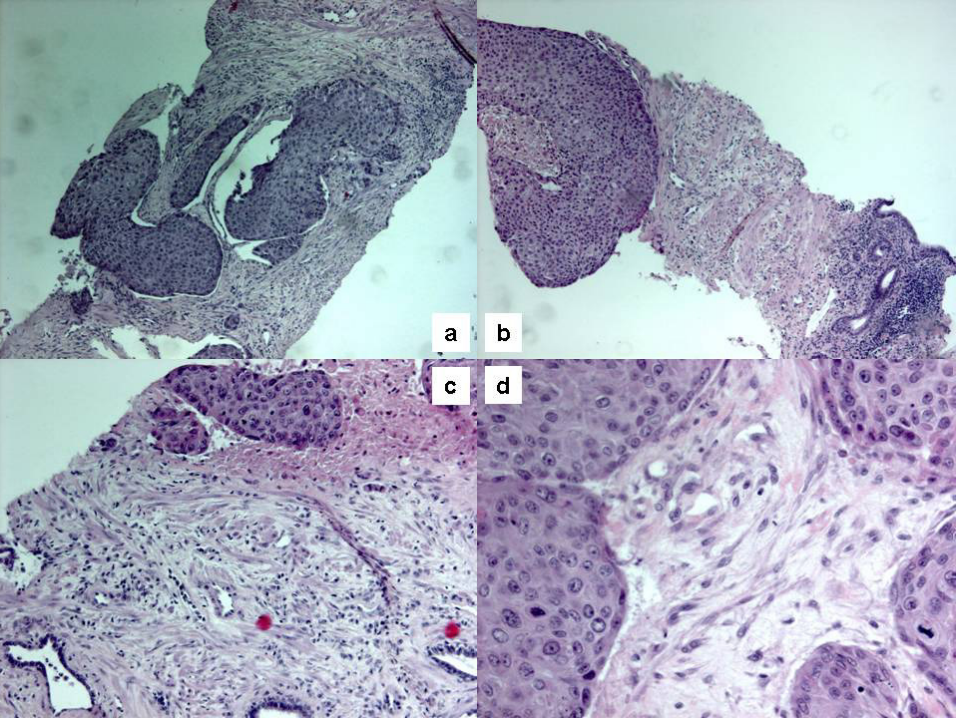
Hematoxilin and eosin-stained sections showing solid sheets of squamous cells infiltrating the right lobe of the prostate (a; original magnification 10×) and the left lobe (b, c; original magnification 10× and 20×, respectively). Mytotic activity may also be observed (d; original magnification 40×).

**Figure 2:**
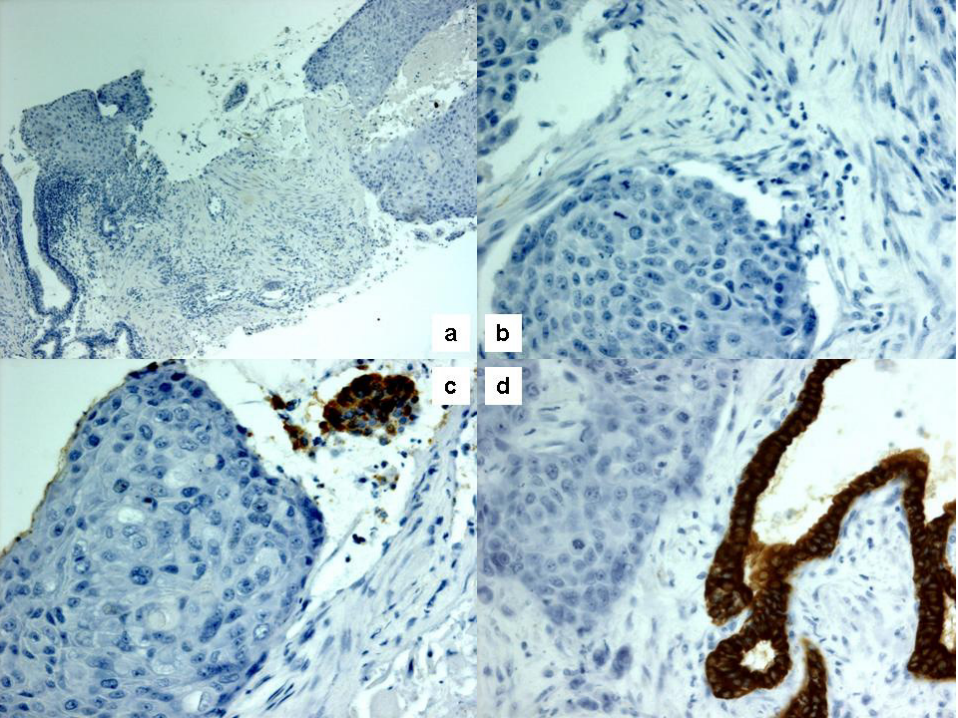
Immunohistochemistry showed negativity to cytokeratin 7 (a; original magnification 10×) and cytokeratin 20 (b; original magnification 40×). Squamous carcinoma cells also stained negative for PSA and PAP, while adjacent remaining glands stained positive (c, d; original magnification 40×).

Immunohistochemically, the PSCC component was non reactive for PSA, PAP and cytokeratin 7 and 20, but stained positive for high molecular weight cytokeratin (Figure 1,2). Considering all the data mentioned above, a diagnosis of AJCC-UICC Stage III (cT3aN0M0) prostate PSCC was then made.

The patient was given 3 courses of chemotherapy (CT) with cisplatin (CDDP) 75 mg/m^2 ^on day 1 and continous infusion 5-fluorouracil (5-FU) 750 mg/m^2 ^on day 1 to 5.

He subsequently underwent a full-course of radiation therapy (RT) using linear accelerator (18 MV photons), with a conventional four-field box technique up to 46 Gy to the whole pelvis followed by a boost dose to the prostate bed of 20 Gy and by an adjunctive dose of 6 Gy to the prostate gland (72 Gy in total). The patient was treated in supine position, with a six-field conformal field arrangement, delivered using three-dimensional conformal RT. Treatment was, generally, well tolerated, with no acute toxicity recorded.

After therapy had been completed, the patient was periodically examinated, with a clinical, radiological and serologic follow-up. He remained free from disease for 5 years, finally relapsing locally within the pelvis and subsequently dying of acute renal failure due to bilateral uretero-hydro-nephrosis in June 2006.

## Discussion

PSCC of the prostate gland is an extremely rare pathological and clinical entity. Its histogenesis remains unclear. Mainly 2 principal origins are presumed for the neoplastic cells: the basal or reserve cells of prostatic acini, as pointed out by Sieracki, and the transitional epithelium lining the urethra or major ducts, as preferred by Kahler and Thompson *et al *[8-10]. Several theories have been proposed in order to explain prostate PSCC histogenesis: 1) a metaplastic transformation of adenocarcinoma cells, 2) a collision-type tumour, with the squamous component developing from metaplastic foci after radiation or hormonal therapy, 3) possible deviation from pluripotent stem cells capable of multidirectional differentiation [11-14]. It has been speculated that the squamous cell component might be derived from squamous metaplasia of acini and ductal elements following radiation or hormonal therapy for prostatic adenocarcinoma [13]. While squamous metaplasia is known for occurring within the prostate gland during chronic prostatitis, around prostatic infarcts and after estrogen therapy or radiation therapy, malignant transformation is rare [8]. Moreover, even though prostate PSCC has been described in association with the use of a luteinising hormone-releasing hormone agonist and flutamide or after seed implantation for adenocarcinoma, all of these explanations could not reach satisfactory evidence [15,16].

Mott suggested strict criteria for the diagnosis of primary PSCC: (i) a clearly malignant neoplasm as judged by invasion, disordered growth and cellular anaplasia; (ii) definite squamous features of keratinization, squamous pearls and/or numerous distinct intercellular bridges; (iii) a lack of any glandular or acinar pattern (such a finding should be interpreted as evidence of adenocarcinoma with squamous metaplasia); (iv) no prior estrogen therapy; (v) an absence of primary squamous cancer elsewhere, particularly within the bladder [4].

A dismal prognosis has been usually associated with PSCC of the prostate (see Table 1 for details). The average survival time has been estimated to be 14 months [6]. As reported by Thompson *et al*, 5 out of 7 patients of their series survived less than 1 year [10]. In this context, scant information is available hitherto concerning the most valid treatment option for prostate PSCC. It does appear feasible that at least some localised tumours can be resected with similar modalities to comparably staged adenocarcinoma of the prostate and long-term survivors in this setting have been reported [10,17-19]. Little *et al *treated 2 patients with an aggressive surgical approach, consisting of radical cysto-prostatectomy and bilateral pelvic lymphadenectomy with an additional total urethrectomy in order to assure negative distal urethral margins: one patient remained free from disease 40 months after initial diagnosis, while the other died of lung metastases 25 months after diagnosis [17]. Gray *et al *performed transpubic radical cysto-prostatectomy with complete urethrectomy and bilateral pelvic node dissection in one patient. Moreover the pubic symphysis was excised and an abdominoperineal resection of the rectum was accomplished with a sigmoid colostomy. Unfortunately, that patient died 6 months after surgery of perineal recurrence [18].

**Table 1:** Overview on clinical reports

**Authors**	**N° of cases**	**Treatment Options**	**Outcome**
**1. Radical surgery reports**			

Baker *et al *and Arnheim [25,26]	4	Orchiectomy/TURP/RP	Survival times: 1 to 3 mo.
Thompson *et al *[10]	7	TURP/Orchiectomy/ES	Survival times: 3 mo.-6 yrs range
			5/7 pts survived less than 1 year
Sieracki [8]	3	RP/RT/ES	Survival times: 8 days-9 yrs range
Gray *et al *[18]	1	RP/Miles	Died 1 year after diagnosis
Corder *et al *[23]	1	TURP/RP/RT/Adriamycin	5 mo. response to CT
Samsonov [31]	3	RP	NA
Al Adnani [32]	2	RP	Not reported
Asuero *et al *[33]	1	RP	Alive 1 year after treatment
Sarma *et al *[35]	1	RCP/TP/SC/PS/Miles	Died 6 mo. after diagnosis of d-MTS
Masuda *et al *[19]	1	RP	Disease-free 6 yrs after surgery
Moskovitz *et al *[6]	1	RP	Died 5 mo. after diagnosis
Little *et al *[17]	2	RCP/PLND/TURP	Disease-free 40 mo. after surgery
		RCP/PLND	Lung MTS 25 mo. after diagnosis
Braslis *et al *[15]	1	RCP	Disease-free 6 mo. after surgery
Okamoto *et al *[7]	1	RP/PLND/MPD	Died 14 mo. after surgery
Imamura *et al *[22]	1	RCP/MPD	Disease-free 5 yrs after surgery
Kanthan *et al *[45]	6	RP/RT/CT	Survival rates from 1 to 13 mo.

**2. Radio-chemotherapy combined modality treatment reports**			

Inaba et al [49]	1	TURP/CDDP/RT	Died 13 months after diagnosis
Kuwahara *et al *[36]	1	TURP/CDDP/PEP/RT	Died 9 mo. after diagnosis
Uchibayashi *et al *[20]	1	TURP/Local RT/CDDP/BLM	Disease-free 21 mo. after diagnosis
Okada *et al *[21]	1	Local RT/CDDP/PEP	Disease-free 18 mo. after diagnosis
Present case	1	RT/CDDP/5-FU	5 yrs disease-free survival

**3. Miscellaneous**			

Ray *et al *[27]	1	RT	Not reported
Kastendieck *et al *[28]	1	TURP/Orchiectomy/ES	Died 2 months after diagnosis
Mott [4]	2	TURP/RT	Died 8 mo. after diagnosis
		Orchiectomy/RT/DES	Died 5 months after diagnosis
Sharma *et al *[29]	1	Orchiectomy	Died during diagnostic procedures
Miller *et al *[16]	1	CDDP/5-FU/Tax	SD for 6 months
Perez Garcia *et al *[37]	1	RT	Alive 5 mo. after treatment
Nabi *et al *[41]	2	TURP/Adriamycin	Died 5 mo. after diagnosis
Goto *et al *[42]	1	MTX/Pirarubicin/CDDP/TPE	Disease-free 12 mo. after therapy
Mayayo *et al *[44]	1	Local RT	Died 7 mo. after diagnosis
John *et al *[47]	1	TURP/RT	Died 24 mo. after diagnosis
Di Pietro *et al *[48]	1	TURP	Died during diagnostic work-up

**4. Anecdotal reports**			

Spagnol [24]	1	None	Died 2 days after diagnosis
Kahaler [9]	6	Not reported	Not reported
Okamura *et al *[30]	1	NA	NA
Wernert *et al *[34]	1	Not reported	Not reported
Ulloa *et al *[38]	1	NA	NA
Rahmanou *et al *[39]	1	Not reported	Not reported
Puyol *et al *[40]	1	None	Local PD 6 mo. after diagnosis
Herrera *et al *[43]	1	None	Died 2 mo. after diagnosis
Mohan *et al *[5]	1	None	Discontinued follow-up
Parwani *et al *[46]	8	Not reported	Survival times: 5–48 mo.

Only pure squamous cell carcinoma of the prostate gland have been included. TURP: transurethral resection of prostate; RP: radical prostatectomy; RT: radiotherapy; CT: chemotherapy; NA: not available (mainly due to articles written in languages others than English); ES: estrogen; DES: diethylstilbesterol; mo.: months; yrs: years; RCP: radical cystoprostatectomy; TP: total penectomy; SC: scrotectomy; PS: pubic symphysectomy; Miles: abdominoperineal resection of the rectum; d-MTS: distant metastases; CDDP: cisplatin; PEP: peplomycin; PLND: pelvic lymphadenectomy; 5-FU: 5-fluorouracil; Tax: paclitaxel; MPD: methotrexate-peplomycin-cisplatin; BLM: bleomycin; MTX: methotrexate; TPE: total pelvic exenteration.

In Japan the longest survivor, who has been described, had a tiny suburethral PSCC; he remained free of recurrence for 6 years, after local excision, as reported by Masuda *et al *[19]. As far as tumour response to therapy is concerned, prostate PSCC is generally refractory to hormonal manipulation, while few cases might be susceptible to CT and RT. Several drugs have been employed, mainly basing on the experience with epithelial tumours located in other anatomical sites. CDDP-based regimens are the most established ones, possibly combined with bleomycin (BLM), peplomycin (PEP) and methotrexate (MTX). CT may be used as a single agent in a metastatic disease setting or in a combined modality approach, expecially in locally advanced disease. In this context, Uchibayashi *et al *achieved a favourable local tumour control for 21 months after trans-urethral prostatectomy by means of local irradiation and, subsequently, intravenous administration of BLM and intra-arterial administration of CDDP [20]. Okada *et al *achieved a 18 months disease-free survival with 50 Gy of pelvic RT plus an additional boost of 10 Gy and systemic administration of PEP and CDDP [21].

Imamura *et al *used CT within an adjuvant setting, after radical cystoprostatectomy with positive surgical margins. The patient received the MPD regimen with CDDP, PEP and MTX. No evidence of recurrence could be detected 5 years after treatment [22].

Corder *et al *treated one patient with pulmonary metastases from prostate PSCC with the administration of adriamycin 20 mg/m2 daily for 3 days, in 21-days cycles, achieving marked regression of pulmonary nodes. Tumour response lasted 5 months [23]. One patient receiving RT survived almost 9 years as reported by Sieracki et al [8].

In conclusion, from this concise overview of all reported cases, it appears evident that no definitive conclusions can be drawn up to now regarding the best available treatment option towards prostate PSCC. Nonetheless, certain features might be noteworthy. In a clinical situation of an organ-confined disease, radical surgical extirpation should be performed. The extent of the surgical procedure remains unclear. Radiation therapy as a single modality treatment in limited-stage disease seems to be an investigational approach. Moreover, the role of RT within an adjuvant setting after surgery has rarely been explored. However, within a context of a locally-advanced disease, RT might be usefull (with doses of at least 66 Gy, deriving data from SCC at other anatomic sites), expecially if combined with chemotherapy, in order to achieve local control with organ functional preservation. The possibility to diminish the probability of systemic spread may also be hypotesised. The most effective drugs to be used have yet to be established. More data are needed in the future, maybe collected in a retrospective multicentric setting or through rare cancer networks, in order to, supposedly, enlighten this subject.

## Financial competing interests

The author(s) declare that they have no competing interests.

## Authors' contributions

FM treated the patient and contributed in the critical revision. PF drafted the manuscript. PC contributed in the acquisition, analysis and interpretation of data. MC treated the patient and contributed in the acquisition, analysis and interpretation of data. MG supplied the pathological specimens. ARF contributed in the critical revision. UR gave final revision and approval. All authors read and approved the final manuscript.
